# Hydroxytyrosol Counteracts Triple Negative Breast Cancer Cell Dissemination via Its Copper Complexing Properties

**DOI:** 10.3390/biology12111437

**Published:** 2023-11-16

**Authors:** Nunzio Perta, Laura Torrieri Di Tullio, Elisa Cugini, Paola Fattibene, Maria Cristina Rapanotti, Ilaria Borromeo, Cinzia Forni, Patrizia Malaspina, Tiziana Cacciamani, Daniele Di Marino, Luisa Rossi, Anastasia De Luca

**Affiliations:** 1 Department of Life and Environmental Sciences, Polytechnic University of Marche, Via Brecce Bianche, 60131 Ancona, Italy; n.perta@pm.univpm.it (N.P.); t.cacciamani@univpm.it (T.C.); d.dimarino@staff.univpm.it (D.D.M.); 2 New York-Marche Structural Biology Center (NY-MaSBiC), Polytechnic University of Marche, Via Brecce Bianche, 60131 Ancona, Italy; 3 Istituto Superiore di Sanità, Core Facilities, Viale Regina Elena, 299, 00185 Rome, Italy; laura.torrieriditullio@guest.iss.it (L.T.D.T.); paola.fattibene@iss.it (P.F.); 4 PhD School in Biochemistry, Department of Biochemical Sciences “A. Rossi Fanelli”, University of Rome “Sapienza”, Viale Regina Elena, 332, 00185 Rome, Italy; 5 Department of Laboratory Medicine, University of Rome Tor Vergata, Viale Oxford, 8, 00133 Rome, Italy; elisa.cgn@gmail.com (E.C.); mariacristina.rapanotti@ptvonline.it (M.C.R.); 6 PhD School in Evolutionary Biology and Ecology, Department of Biology, University of Rome Tor Vergata, Via della Ricerca Scientifica 1, 00133 Rome, Italy; ilaria18scv@hotmail.it; 7 Department of Biology, University of Rome Tor Vergata, Via della Ricerca Scientifica 1, 00133 Rome, Italy; forni@uniroma2.it (C.F.); patrizia.malaspina@uniroma2.it (P.M.); luisa.rossi@uniroma2.it (L.R.); 8 Neuronal Death and Neuroprotection Unit, Department of Neuroscience, Mario Negri Institute for Pharmacological Research-IRCCS, Via Mario Negri 2, 20156 Milano, Italy

**Keywords:** polyphenols, copper, epithelial to mesenchymal transition, AKT, copper complex, EPR, molecular modeling

## Abstract

**Simple Summary:**

Several naturally occurring substances, known as polyphenols, may be found in a variety of plant-based foods and drinks, including fruits, vegetables, whole grains, tea, and red wine. Several studies have shown that these molecules have many beneficial effects on human health, particularly in their ability to counteract tumor growth thanks to their capacity to dampen inflammatory processes. Furthermore, polyphenols can form complexes with certain metals, such as copper. This property is of great importance considering that copper is closely involved in both the early stages and progression of cancer. In this study, we investigated the capacity of hydroxytyrosol, a derivative of oleuropein found in the leaves and fruits of the *Olea europaea* plant, to complex with copper and, consequently, to counteract the progression of triple-negative breast cancer. We chose this type of cancer as it is the most aggressive subtype due to the lack of specific receptors, which results in the absence of targeted oncological therapy. Our results demonstrate that hydroxytyrosol forms a complex with copper, resulting in the reduction of its content within the triple-negative breast cancer cell, consequently reducing its aggressiveness and, eventually, its ability to form metastases.

**Abstract:**

Polyphenols have gained increasing attention for their therapeutic potential, particularly in conditions like cancer, due to their established antioxidant and anti-inflammatory properties. Recent research highlights their ability to bind to transition metals, such as copper. This is particularly noteworthy given the key role of copper both in the initiation and progression of cancer. Copper can modulate the activity of kinases required for the epithelial–mesenchymal transition (EMT), a process fundamental to tumor cell dissemination. We have previously demonstrated the copper-binding capacity of oleuropein, a secoiridoid found in *Olea europaea*. In the present study, we investigated the effect of hydroxytyrosol, the primary oleuropein metabolite, on the metastatic potential of three triple-negative breast cancer cell lines (MDA-MB-231, MDA-MB-468, and SUM159). We found that hydroxytyrosol modulated the intracellular copper levels, influencing both the epithelial and mesenchymal markers, by downregulating copper-dependent AKT phosphorylation, a member of the EMT signaling cascade, through Western blot, RT-qPCR, and immunofluorescence. Indeed, by optical spectra, EPR, and in silico approaches, we found that hydroxytyrosol formed a complex with copper, acting as a chelating agent, thus regulating its homeostasis and affecting the copper-dependent signaling cascades. While our results bring to light the copper-chelating properties of hydroxytyrosol capable of countering tumor progression, they also provide further confirmation of the key role of copper in promoting the aggressiveness of triple-negative breast cancer cells.

## 1. Introduction

Polyphenols are a group of naturally occurring compounds found in various plant-based foods, such as fruits, vegetables, whole grains, and beverages like tea and red wine [1]. They have gained considerable attention for their potential health benefits and their ability to counteract cancer onset and progression [2]. In particular, polyphenols exhibit strong antioxidant properties, which enable them to scavenge harmful free radicals and reduce oxidative stress [3,4]. By neutralizing free radicals, polyphenols protect cells from oxidative damage and potentially inhibit tumor breast cancer initiation and growth [5,6]. Furthermore, they exhibit anti-inflammatory properties, and a significant correlation exists between chronic inflammation and the development of cancer [7,8]. In light of all these properties, research has also focused on studying polyphenols’ effects on triple-negative breast cancer (TNBC), an aggressive subtype of breast cancer characterized by a lack of expression of the estrogen receptor (ER), the progesterone receptor (PR), and human epidermal growth factor receptor 2 (HER2) [9,10]. Indeed, it has been shown that polyphenols can modulate inflammatory pathways and suppress the production of pro-inflammatory molecules [4,5] leading to the reduction of the growth and progression of TNBC cells [10,11,12]. Beside their well-known anti-oxidant and anti-inflammatory properties, natural compounds are also able to influence various stages of the cell cycle, including cell proliferation, cell differentiation, and programmed cell death (i.e., apoptosis) [13,14,15,16].

In addition to these anti-cancer and anti-inflammatory features, polyphenols can form complexes with the transition metal copper, functioning as metal-chelating agents [17]. The ability of polyphenols to complex copper is significant because copper plays a crucial role in cancer progression through various mechanisms. Copper is involved in angiogenesis by stimulating the production of vascular endothelial growth factor (VEGF), a key regulator of angiogenic mechanisms [18]. Of note, this transition metal is also required during extracellular matrix (ECM) remodeling and the epithelial to mesenchymal transition (EMT), the critical steps of cancer cell dissemination. Indeed, all these processes are coordinated by copper-dependent enzymes: (i) the lysyl oxidase family (LOXs and LOXLs), which promotes ECM cross-linking and stiffness; (ii) the phosphatidylinositol 3-kinase (PI3K)/RAC-alpha serine/threonine-protein kinase (AKT) pathway, whose dysregulation is associated with cancer cell survival, the EMT, and resistance to apoptosis; and (iii) the mitogen-activated protein kinase MEK1/2 which, in turn, phosphorylates extracellular signal-regulated kinase 1/2 (ERK1/2), driving cancer cells through proliferation and EMTs [19]. Considering the central role of copper in the development and progression of cancer, the potential use of polyphenols to complex this metal, limiting its bioavailability, deserves further investigation.

To date, several polyphenols have been identified for their ability to bind copper ions. Among them, quercetin, a flavonoid widely present in various fruits, vegetables, and beverages [20,21]; epigallocatechin-3-gallate (EGCG), a major catechin found in green tea, has shown the ability to bind copper ions and form complexes [22]; resveratrol, a polyphenol found in grapes, berries, and wine [23]; and curcumin, derived from turmeric [24]. These are just a few examples of polyphenols known to bind copper ions. It is important to note that the specific binding affinities and mechanisms may vary among different polyphenols and require further investigation in specific contexts.

Our group has demonstrated that also oleuropein (Ole), a phenolic compound belonging to the secoiridoid group, present in the leaves and fruits of the olive tree *Olea europaea*, can complex copper [25]. Ole has shown an inhibitory effect on the cell cycle, proliferation, and migration, as well as the promotion of apoptosis, in several TNBC cell lines: in MDA-MB-468 and MDA-MB-231 cells, Ole induced cell growth inhibition and apoptosis by inducing S-phase cell cycle arrest [26,27]. Additionally, Ole upregulated the expression of several genes involved in the apoptosis of TNBC cells and it was shown to increase the expression of pro-apoptotic genes and tumor suppressor miRNAs and decrease the expression of anti-apoptotic genes and oncomiR [28].

Hydroxytyrosol (3,4-dihydroxyphenylethanol) (HDT) is a phenolic alcohol present in olive oil and is the major metabolite of Ole. As for all the previously cited polyphenols, its beneficial properties for human health are strongly linked to the molecule’s ability to eliminate free radicals and reactive oxygen species (ROS), as well as to activate the body’s antioxidant systems. HDT has shown antitumor activity through different mechanisms [27]. Interestingly, it has been demonstrated that, in the human breast cancer cell line MCF 7, the antioxidative properties of HDT are particularly effective in hypoxic conditions, even if the authors do not completely clarify the mechanism of HDT action on oxidative stress [29]. Additionally, HDT has been shown to modulate MDA-MB-231 TNBC cell line migration and invasion, in a dose-dependent manner, by attenuating the EMT [30]. These effects were mediated by the dual inhibition of the Wnt/β-catenin and transforming growth factor beta (TGFβ) signaling pathways [31]. Additionally, it has been found that in rat mammary tumors, HDT treatment reduced the expression of the EMT transcription factor (EMT-TF) Snail family transcriptional repressor 1 (SNAI1) and Snail family transcriptional repressor 2 (SNAI2) [32]. Furthermore, HDT rich extract from olive leaves modulated cell cycle progression in MCF-7 human breast cancer cells, by promoting the cell cycle blocking in the G1 phase following the down-expression of peptidyl-prolyl cis–trans isomerase (Pin1), which in turn decreased the level of cyclin D1 [33]. It has also been proposed that HDT, together with Ole, inhibits migration by inducing autophagy in triple-negative (i.e., MDA-MB-231) and ER-positive (i.e., MCF-7) breast cancer cell lines [34].

Given all these appealing properties of HDT, in the present study, we aim to understand whether its ability in counteracting the EMT could rely on the formation of a copper–HDT complex impairing the copper-dependent signaling fueling the MDA-MB-231 and MDA-MB-468 TNBC cells dissemination.

## 2. Materials and Methods

### 2.1. Chemicals

HDT, CuSO_4_, MTT (3-(4,5-dimethylthiazol-2-yl)-2,5-diphenyl tetrazolium bromide), 4% paraformaldehyde, Triton X-100, crystal violet, EDTA, EGTA, and NP-40 were purchased from Merck Life Science S.r.l, Milan, Italy.

### 2.2. Cell Cultures and Treatments

The TNBC cell lines MDA-MB-231, MDA-MB-468, and SUM159 cells were purchased from the American Type Culture Collection (ATCC, Manassas, VA, USA). The cells were grown in sterile 75 cm^2^ flasks, at 37 °C, in a humidified atmosphere of 5% CO_2_, in DMEM culture medium with stable L-glutamine (EuroClone, Milan, Italy), 10% (*v*/*v*) fetal bovine serum (FBS, EuroClone), and 1% of a mixture of antibiotics (penicillin and streptomycin 100 U/mL). The cells were grown to 90% confluence and, subsequently, the medium was removed from the flask and washing with DPBS (EuroClone) was carried out to remove any medium residues. Afterwards, cells were seeded in 6-wells plates at a density of 0.3 × 10^4^ cells/cm^2^ for MDA-MB-231 and MDA-MB-468, and 0.1 × 10^4^ cells/cm^2^ for SUM159. Approximately 8 h after seeding, the 10% FBS DMEM medium was removed and replaced with DMEM containing 1% FBS. Following 24 h of serum deprivation, cells were treated with 100 μM HDT for up to 72 h.

### 2.3. Cell Viability Assessment by MTT Assay

To evaluate the effect of HDT on cell proliferation, TNBC cells were seeded in sterile 96-wells plates at a density previously reported. Twenty-four hours after seeding, cells were treated with increasing concentrations of HDT (ranging from 0.5 to 300 5 μM), for 72 h. HDT was dissolved in ddH_2_O, stock solution 100 mM. Then, the culture medium of each well was replaced with 200 μL of MTT (0.5 mg/mL) diluted in complete DMEM medium and incubated for up to 4 h, following manufacturer protocol. The absorbance of formazan was read at 590 nm in a multi-well plate reader (Infinite M^®^ Plex, Tecan Life Sciences, Männedorf, Switzerland).

### 2.4. Western Blot Analysis

After treatments, cells were harvested, washed in PBS, and lysed in RIPA buffer (10 mM Tris–HCl pH 7.4, 1 mM EDTA, 1 mM EGTA, 1% NP-40, 30 mM NaCl, and protease inhibitor cocktail), following 20 min incubation on ice. The samples were centrifuged at 1000× *g*, 20 min, at 4 °C. The protein concentration of the supernatant was determined using the Lowry colorimetric assay (DCTM ProteinAssay, BioRad, Hercules, CA, USA). The samples were diluted in 3× Blue Loading Buffer plus DTT (Cell Signaling Technology, Inc., Danvers, MA, USA) and denatured at 95 °C for 5 min. Proteins (30 μg) were separated on 8, 10, or 12% SDS-polyacrylamide gel and transferred to a nitrocellulose membrane (0.45 μm; BioRad). Table 1 reports primary antibodies used for immunodetection, as well as their dilutions. Anti-rabbit or anti-mouse secondary antibodies (Cell Signaling Technology, Inc.) were revealed with the ECL (ECL Prime Western Blotting Reagent, Cytiva Europe GmbH, Freiburg, Germany) by the ImageQuant LAS 4000 (Fuji Film, Tokyo, Japan). Densitometric analyses were performed through the ImageJ 1.5 software (NIH, Bethesda, MD, USA). Vinculin, actin, or tubulin were used as loading control.

### 2.5. Immunofluorescence Analysis

MDA-MB-231, MDA-MB-468, and SUM159 cells were seeded on a round coverslip (12 mm). The day after seeding, cells were treated with 100 μM HDT for up to 72 h. At the end of the treatment, the medium was removed, and cells were washed in PBS and fixed by incubation in a solution of PBS/4% paraformaldehyde (*v*/*v*), for 10 min, at room temperature. Cells were then permeabilized following 20 min incubation in PBS/0.1% Triton X-100 (*v*/*v*) at room temperature. Subsequently, cells were incubated in PBS/5% FBS, for 1 h, at room temperature. Upon two washes in PBS, the coverslips were incubated with the rabbit polyclonal anti-fibronectin antibody (1:200), or mouse monoclonal anti-occludin (1:50), for 18 h, at 4 °C. Afterwards, coverslips were incubated with the anti-rabbit IgG AlexaFluor 488 or the anti-mouse IgG AlexaFluor 488 (1:1000, Invitrogen, Waltham, MA, USA) for 1 h, in a humid chamber, in the dark, and at room temperature. To label cells, cytoskeleton phalloidin-TRITC (1:1000, Merck Life Science, #P1951) was added to the secondary antibodies mixture. At the end of the incubation, coverslips were mounted using 5 μL of ProLong Gold Anhtifade Reagent with DAPI (Invitrogen). Image acquisition was performed with a Leica DMR450 FX fluorescence microscope (Milan, Italy), equipped with a DFC350 FX camera, 40× magnification.

### 2.6. Wound Healing Assay

TNBC cells were seeded in a 6-well plate. Seventy-two hours after seeding, a scratch was made on the cell monolayer with the tip of a sterile pipette along the central axis of the well. After 2 washes with PBS, DMEM without FBS was added in the absence or in the presence of 100 μM HDT. Cell migration to restore monolayer integrity was monitored over time by acquiring images under an inverted microscope (Nikon Eclipse Ts2, Amstelveen, The Netherlands), 10× magnification, up to 48 h. The closure of the wound area was analyzed through the ImageJ software. Cell migration was expressed as the percentage of scratch width that heals as a function of time, examining its extent in at least 5 random fields, under the microscope, for each individual condition and observation time.

### 2.7. TNBC Aggressiveness Evaluation by Boyden Chamber Migration and Invasion Assay

Cells were seeded in the upper compartment of transwell inserts (8 μM pore size, Corning, NY, USA) in a 24-wells plate, at a density of 0.5 × 10^4^ cells/well, in FBS-free medium, in the presence or absence of 100 μM HDT. Complete medium containing 10% FBS was added to the bottom. After 48 h of incubation at 37 °C in 5% CO_2_, the medium was removed from both compartments, the transwells were washed with PBS, and the TNBC cells adherent to the underside of the microporous septum were fixed and permeabilized with cold 70% ethanol, washed in PBS, and stained with 0.25% crystal violet. Migrated cells were visualized with an inverted microscope (Nikon Eclipse Ts2), 10× magnification. Images were acquired by a digital camera (TP1080HDMI, Nikon, Amstelveen, The Netherlands). At least four representative fields were chosen for each condition and cells were counted using the ImageJ program.

For the invasion assay, we used the Corning BioCoat Matrigel Invasion Chambers (Corning, NY, USA) and followed the same procedure of the migration assay.

### 2.8. Real-Time-Quantitative PCR (RT-qPCR)

Total RNA was extracted from TNBC cells using TRIzol (Invitrogen), and 2.5 μg of RNA were reverse transcribed using reverse transcriptase MMLV (Moloney murine leukemia virus) (Promega Corporation, Madison, WI, USA). Subsequently, Real-Time PCR was performed in a QuantStudio 3 thermocycler (Thermo Fisher Applied Bioystem, Rome, Italy) in 20 μL of final volume containing 10 ng of cDNA, 5 μM of each prime (reported in Table 2), and 50% of SYBR green (Kapa SYBR Fast qPCR kit; Roche Kapa Biosystems, Wilmington, MA, USA). The relative expression of each gene was evaluated using the 2^−ΔCt^ method, i.e., comparing the difference between the Ct values of the gene under examination and those of the β-actin gene in the treated sample compared to the control sample.

### 2.9. UV-VIS and EPR Spectroscopy

To study the complex formation between HDT and copper, optical spectra were recorded using a Cary 4000 UV/Vis dual-beam spectrophotometer (Agilent Technologies, Santa Clara, CA, USA). HDT (100 mM stock solution) as well as CuSO_4_ (100 mM stock solution) were dissolved in ddH_2_O. HDT and CuSO_4_ were added to cuvettes with different optical paths, according to the final concentrations reported in the figure legends, and at the reciprocal molar ratios 1:1, 2:1, and 4:1. Optical spectra were recorded in the range of 200 to 800 nm. Continuous wave electron paramagnetic resonance (CW-EPR) measurements were performed using a Bruker Elexsys E-500 X-band (9.8 GHz) (Bruker, Billerica, MA, USA) spectrometer equipped with a Bruker 3122SHQE resonator. Spectral acquisition parameters were: 100 kHz modulation frequency; 1 mT modulation amplitude; 10 mW microwave power; 330 mT center magnetic field; 60 mT field scan range; and 1024 data points. All EPR measurements were performed at room temperature with HDT:copper complex solution inserted in a Teflon capillar tube placed in Suprasil^®^ EPR tubes of 3 mm internal diameter (ATS Life Sciences Wilmad, Vineland, NJ, USA). Samples were prepared at different HDT:copper molar ratios (1:1, 2:1, 4:1) as phosphate buffered saline solution, i.e., at physiological pH, in order to compare the results obtained in the in-cell studies. Aqueous stock solutions were used to ensure the correct stoichiometric ratio. The EPR spectra were simulated using the Matlab toolbox Easyspin v. 6.0.0-dev.51 [35].

### 2.10. Molecular Modeling of the HDT–Copper Coordination Complexes

We conducted a computational investigation to explore the potential complexation of Copper (Cu) with HDT as a metal-coordinating ligand (CHEMBL ID: 1950045). The three-dimensional (3D) structure of HDT, with optimized geometry, was calculated using the B3LYP/6-31G (d, p) DFT method via Automated Topology Builder 3.0 webserver (Molecular ID: 366570) [36] (Supplementary Figure S1a,b). For the chelation models, we employed the Avogadro 1.2.0 software [37] using a Cu (II) atom as the central coordination metal, along with two or four HDT molecules. The resulting complexes were refined using the Auto-Optimize tool within Avogadro. To perform the refinement, we employed the Universal Force Field (UFF) and applied the steepest descent algorithm with 1000 steps [38,39]. The UFF reproduces the molecular features of all periodic table elements and works well with inorganic and organometallic materials [40,41]. In the first run, we imposed distance constraints of 1.9 Å and 2.1 Å for the equatorial and axial O donors, respectively [42]. Finally, we released all the constraints and ran the Auto-Optimize tool once again until it reached the lowest potential energy (in kJ/mol) for both the complexation complexes [43,44]. We used ChimeraX [45] to generate the figures, which proved to be an excellent tool for molecular illustration [46].

### 2.11. Statistical Analysis of Data

Data presented are expressed as means ± standard error of the mean (SEM). The significance of the observed differences was evaluated using the GraphPad Prism 8.0 software (GraphPad Software, San Diego, CA, USA); a *p*-value < 0.05 was considered significant (* *p* < 0.05; ** *p* < 0.01; *** *p* < 0.001; *** *p* < 0.0001).

## 3. Results

### 3.1. HDT Treatment of TNBC Cells Modulates the Levels of Intracellular Copper Sensors CCS and CcO

**Figure 1:**
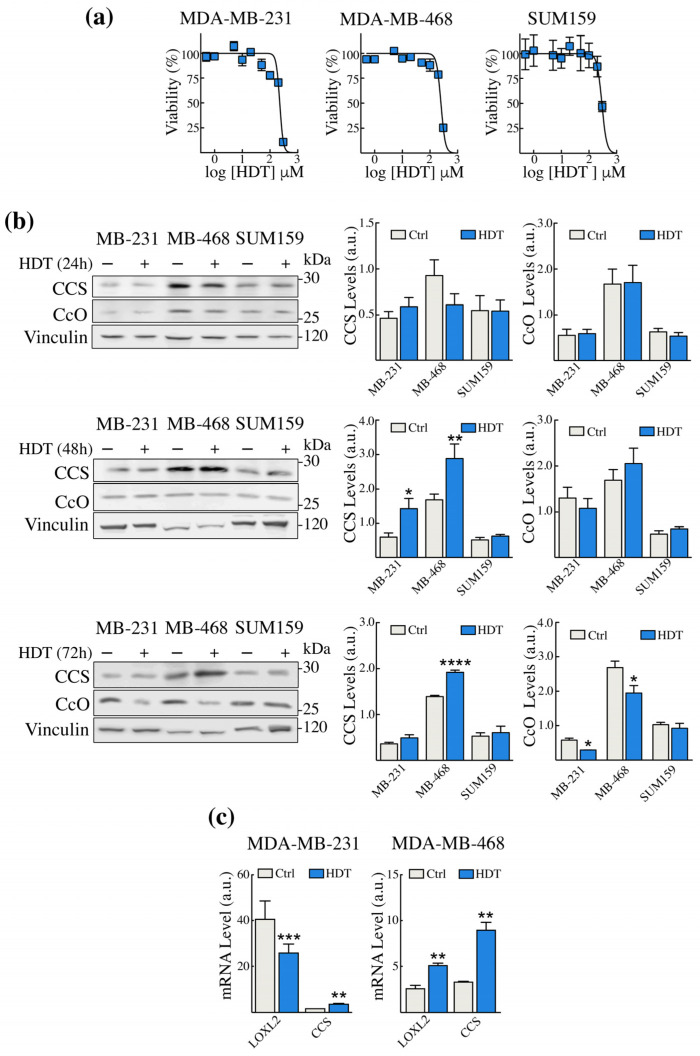
HDT treatment modulates the intracellular copper homeostasis in TNBC cells. (**a**) TNBC cells were treated with increasing concentrations of HDT for 72 h. (**b**) Cells were treated with 100 μM HDT for 24, 48, and 72 h. CCS and CcO Western blot analyses (left panels) and the corresponding densitometric analysis (right panels) were performed. Vinculin was used as a loading control. One representative blot is shown for each antigen out of at least 4 different experiments giving comparable results. (**c**) The level of the CCS and LOXL2 transcripts in MDA-MB-231 and MDA-MB-468 cells were evaluated by RT-qPCR analysis following 24 h exposure to 100 µM HDT. Data are presented as a mean ± SEM (*n* ≥ 3, Student’s *t*-test, * *p* < 0.05, ** *p* < 0.005, *** *p* < 0.0005; **** *p* < 0.0001).

To verify the effect of HDT on TNBC cell lines’ viability, we performed the MTS assay on the MDA-MB-231, MDA-MB-468, and SUM 159 TNBC cells. The MDA-MB-231 cells were the most sensitive to HDT, with an IC_50_ of 230 μM (Figure 1a, left panel). In contrast, the SUM159 cells proved to be more resistant to the action of HDT with an IC_50_ value of 300 μM (Figure 1a, right panel). Based on these results, we chose 100 μM HDT for the subsequent experiments, which is the highest concentration showing the least toxicity.

To assess the impact of HDT treatment on intracellular copper homeostasis, we monitored, by Western blot analysis, the levels of the copper chaperone for superoxide dismutase (CCS) and of the subunit II of the complex IV of the mitochondrial respiratory chain, cytochrome c oxidase (CcO), which are well-established readouts of copper bioavailability. Indeed, CCS is known to be induced in copper depletion conditions, whereas CcO undergoes degradation [47]. We treated cells with HDT and tested the levels of CCS and CcO up to 72 h (Figure 1b). We found that CCS increased significantly both in MDA-MB-231 and in MDA-MB-468 cells after 48 h of HDT treatment (Figure 1b, central panel), and remained significantly elevated even at 72 h in MDA-MB-468 (Figure 1b, bottom panel). This increase at 72 h was mirrored by a decrease in CcO in both the cell lines (Figure 1b, bottom panel). On the contrary, in the SUM159 cell line, no change was observed at any time of exposure. These results suggested that HDT treatment reduced copper bioavailability in MDA-MB-231 and MDA-MB-468 cells, but not in SUM159, at least under our experimental conditions. For this reason, the SUM159 cell line will not be considered for the subsequent experiments.

We further confirm the modulation of CCS in MDA-MB-231 and MDA-MB-468 by assessing its transcript level by RT-qPCR (Figure 1c), following 24 h of HDT treatment. Moreover, we assessed the mRNA level of another copper-dependent enzyme whose activity contributes to ECM remodeling, lysyl oxidase like-2 (LOXL2). We found that, in MDA-MB-231 LOXL2, levels were downregulated upon HDT treatment, in contrast to what was observed in MDA-MB-468.

### 3.2. The Epithelial/Mesenchymal Phenotype of TNBC Cells Is Strongly Affected by HDT

**Figure 2:**
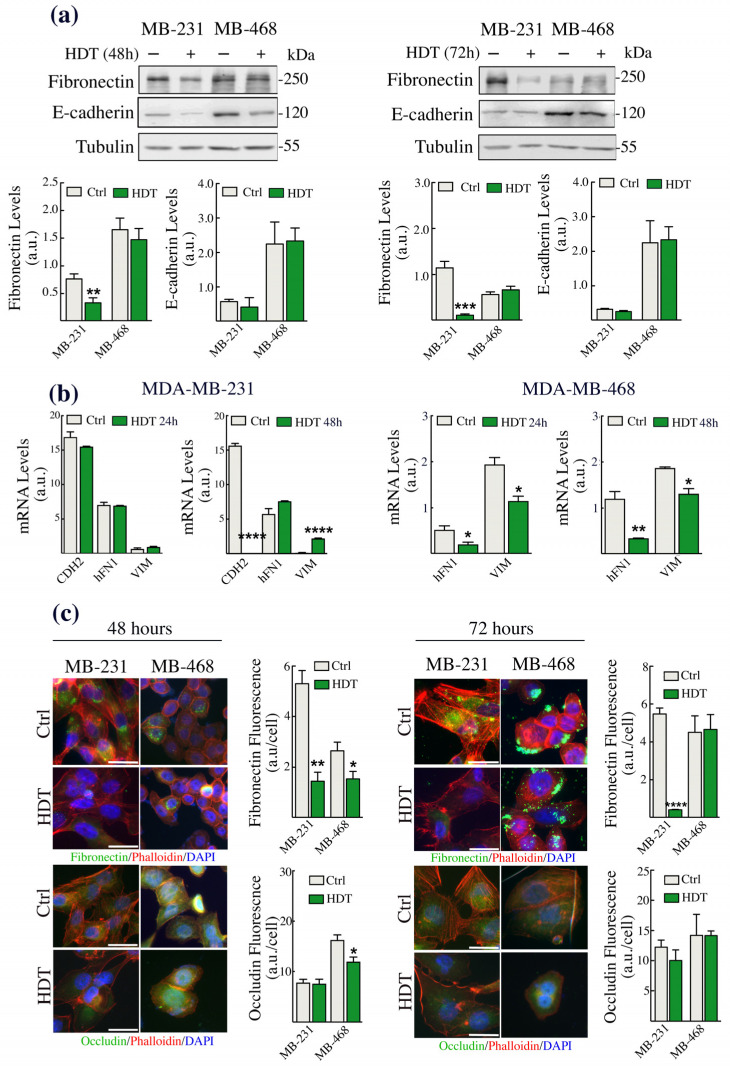
EMT hallmarks change upon HDT treatment of both MDA-MB-231 and MDA-MB-468 cells. (**a**) Western blot analysis of E-cadherin and fibronectin levels in MDA-MB-231 and MDA-MB-468 cells following 48 h (left panel) and 72 h (right panel) exposure to 100 µM HDT and their corresponding densitometric analyses (bottom panels). Twenty micrograms of proteins were loaded on each lane. Tubulin was used as loading controls. Representative blots are shown from at least 4 different experiments giving comparable results. (**b**) The level of the *CDH2*, *hFN1*, and *VIM* transcripts in MDA-MB-231 and of the *hFN1* and *VIM* in MDA-MB-468 cells were evaluated by RT-qPCR analysis following 24 and 48 h exposure to 100 µM HDT. (**c**) Immunofluorescence detection of fibronectin and occludin in MDA-MB-231 and MDA-MB-468 48 h (left panels) and 72 h (right panels) after HDT exposure with 100 µM HDT and their corresponding signal analysis. Representative images are shown from at least 4 different experiments showing comparable results. Scale bar: 125 µm. Data are presented as a mean ± SEM (*n* ≥ 3, Student’s *t*-test, * *p* < 0.05, ** *p* < 0.005, *** *p* < 0.0005, **** *p* < 0.0001).

**Figure 3:**
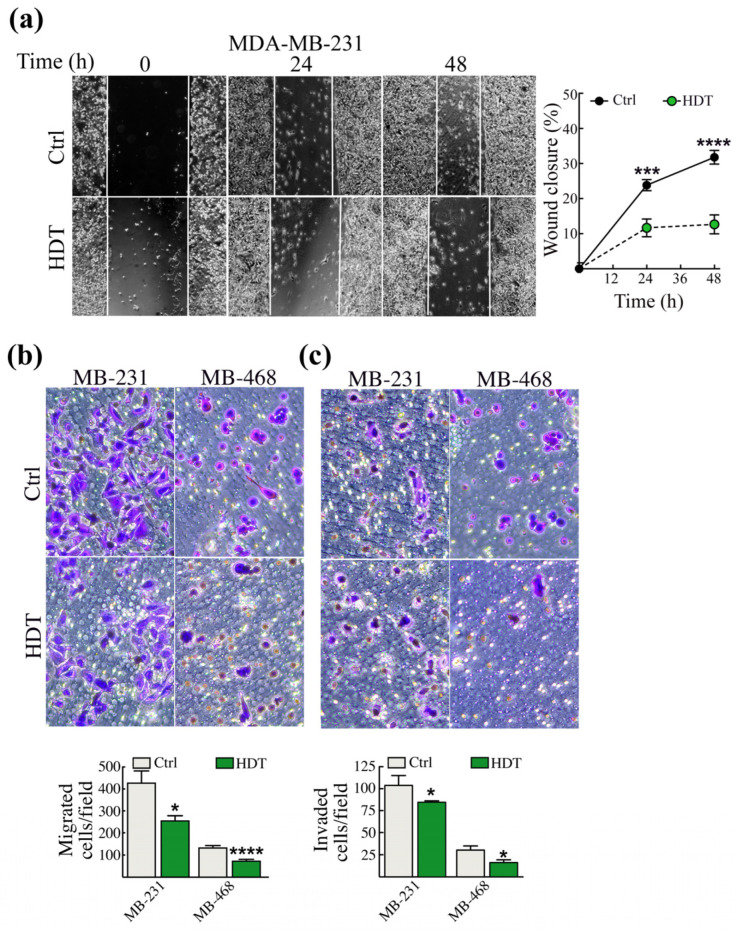
HDT treatment reduces TNBC cells’ aggressiveness to 48 h. (**a**) A single scratch was made in the center of the cell monolayer and the wound closure areas visualized under an inverted microscope with 10× magnification (left panels). Cell motility was quantified by measuring the distance between the invading front of cells in at least 5 random selected microscopic fields for each single condition and time point (right panel). Data are shown as mean ± SEM, *n* ≥ 3, Student’s *t*-test, *** *p* < 0.0005; **** *p* < 0.0001. MDA-MB-231 and MDA-Mb-468 cells were assayed for in vitro migration using a Boyden chamber. After 48 h of exposure to 100 µM HDT, (**b**) migrated and (**c**) invaded cells were stained with crystal violet and counted. One representative phase contrast image (10× magnification) is shown, out of at least three independent experiments. Data are shown as mean ± SEM, *n* ≥ 3, Student’s *t*-test, * *p* < 0.05, **** *p* < 0.0001.

Recent studies highlighted the role of copper both as an allosteric regulator and as the cofactor of enzymes involved in the epithelial to mesenchymal transition (EMT) [48]. Thus, since HDT appeared to modulate the intracellular copper bioavailability, we monitored possible alterations in the expression of EMT hallmarks. Specifically, we assessed, by Western blot analysis, the levels of the epithelial markers E-cadherin or occludin and of the mesenchymal protein fibronectin (Figure 2). Only in MDA-MB-231 cells we found a significant decrease of fibronectin levels at 48 and 72 h of HDT incubation (Figure 2a), whereas we did not observe any variation in the level of E-cadherin in both the cell lines. We further investigated, by RT-qPCR, the modulation of the mRNA levels of *CDH2*, *VIM*, and *hFN1*, the genes coding for the mesenchymal proteins N-cadherin, vimentin, and fibronectin, respectively (Figure 2b). In the MDA-MB-231 cells, we did not observe any alteration of these mRNAs following 24 h of HDT treatment, whilst we found a strong downregulation of the mRNA levels of *CDH2* and an upregulation of *VIM* mRNA upon 48 h of HDT incubation (Figure 2b left panes). The absence of an HDT effect on the *hFN1* mRNA, in contrast to what was observed by Western blot on the fibronectin levels, suggests a possible alteration in the mRNA or protein stability [49]. Interestingly, the MDA-MB-468 cell line did not express *CDH2* (Figure 2b, right panels). However, we found a significant reduction in the mRNA levels of both *hFN1* and *VIM* following 24 and 48 h of treatment with HDT (Figure 2b). Considering the controversial results regarding fibronectin obtained in both the cell lines through Western blot and RT-qPCR, we proceeded with the immunofluorescence analysis of fibronectin and, in addition, we evaluated the levels of the epithelial marker occludin (Figure 3c) in both MDA-MB-468 and MDA-MB-231 cells. The data obtained confirmed the strong downregulation of fibronectin previously obtained by Western blot analysis in MDA-MB-231 and the fibronectin decrease found by RT-qPCR in MDA-MB-468. Moreover, we observed a slight downregulation of occludin in MDA-MB-468 cells.

### 3.3. The Modulation of EMT Hallmarks Prompted by HDT Reflects a Reduced Aggressiveness of TNBC Cells

The induction of EMT signaling is necessary to trigger cancer cell dissemination to distant organs. Thus, we investigated whether the reduction of the mesenchymal markers, observed upon HDT treatment in MDA-MB-231 cells, was reflected in the reduction of TNBC aggressiveness. To this extent, we performed the wound healing assay (Figure 3a). HDT treatment significantly slowed down the wound closure, which stopped at 10% after 48 h, while untreated cells reached about 30% closure (Figure 3a). It was not possible to perform the wound healing assay with the MDA-MB-468 cell line because the cells are prone to detach once confluence is reached.

The reduction of the aggressiveness of MDA-MB-231 was strengthened by the evaluation of TNBC migration/invasion using the Boyden Chamber assay and we performed this experiment also in the MDFA-MB-468 cells. We found a significant decrease of migration in both the cell lines (Figure 3b) following 48 h incubation with HDT. Additionally, upon HDT incubation, the invasive properties of the above reported cell lines were also significantly reduced (Figure 3c).

### 3.4. HDT Triggers EMT by Modulating the Phosphorylation of the Copper-Dependent Kinase AKT

**Figure 4:**
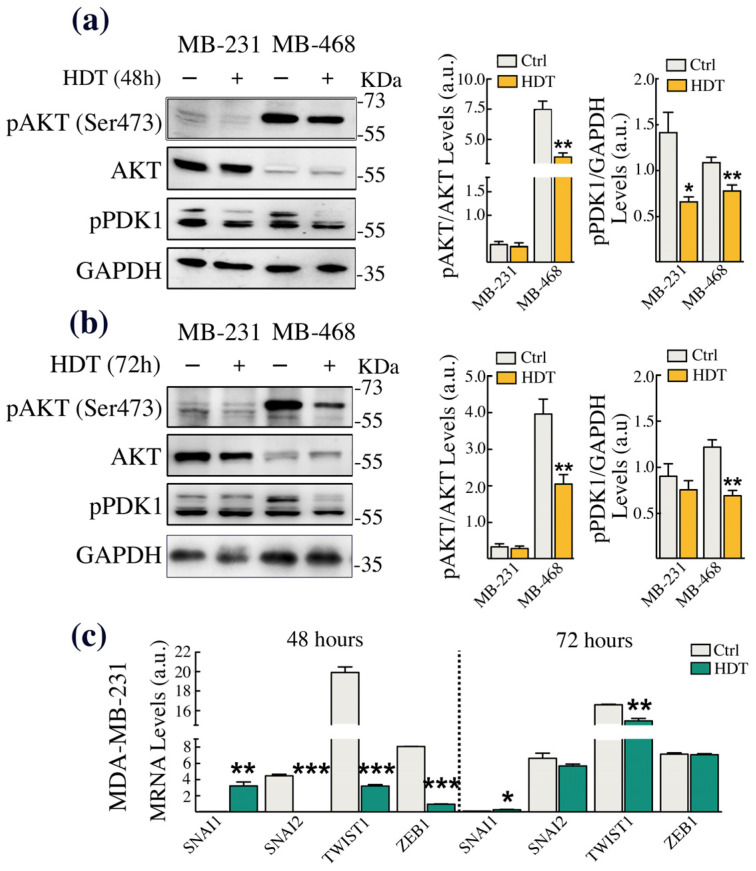
HDT treatment downregulates the copper-dependent phosphorylation of AKT, also affecting the levels of EMT-TFs. MDA-MB-231 and MDA-MB-468 cells were treated with 100 μM HDT for (**a**) 48 h or (**b**) 72 h and analyzed by Western blot for changes in pAKT (Ser473) levels, AKT, and pPDK1. GAPDH was used as a loading control. The respective densitometric analyses are shown in the right panels. (**c**) Transcript levels of the EMT-TFs SNAI1, SNAI2, TWIST1, and ZEB1 measured by RT-qPCR in MDA-MB-231 cells treated with 100 μM HDT for 48 or 72 h. Data are presented as mean ± SEM (*n* ≥ 3, Student’s *t*-test, * *p* < 0.05; ** *p* < 0.005; *** *p* < 0.0005).

It is well established that copper is an allosteric cofactor of kinases participating in the EMT process. Besides the canonical EMT cascade, which occurs upon the engagement of the TGFβRI/II and the recruitment of the SMADs protein family members, copper-dependent MAPK signaling is also required for the non-canonical initiation of the EMT [19]. This includes AKT, which can be phosphorylated in the Tyr308 residue by the copper-dependent kinase PDK1 [50] or, as we recently demonstrated, at Ser473, again in a copper-dependent fashion [51]. Thus, to investigate whether the HDT-induced alteration of the EMT hallmarks, resulting in a reduction of TNBC aggressiveness, was related to the modulation of AKT phosphorylation, we performed Western blot analysis. The data obtained demonstrated that HDT treatment of MDA-MB-468, up to 72 h, significantly reduced the phosphorylation of both AKT (in Ser473) and of PDK1, suggesting the further reduction of AKT phosphorylation in the Tyr308 residue (Figure 4a,b). In contrast, in MDA-MB-231, we found a downregulation of PDK1 phosphorylation at 48 h of HDT incubation. To assess whether this downregulation in MDA-MB-231 was sufficient to induce the anti-metastatic effects of HDT, we perform an RT-qPCR analysis of EMT-TFs SNAI1, SNAI2, TWIST1, and ZEB1 at 48 and 72 h of treatment with HDT. The results obtained showed a strong downregulation of SNAI2, TWIST1, and ZEB1 at 48 h. For TWIST1, the reduction persisted up to 72 h of HDT treatment (Figure 4c).

### 3.5. Formation of HDT: Copper Complex

**Figure 5:**
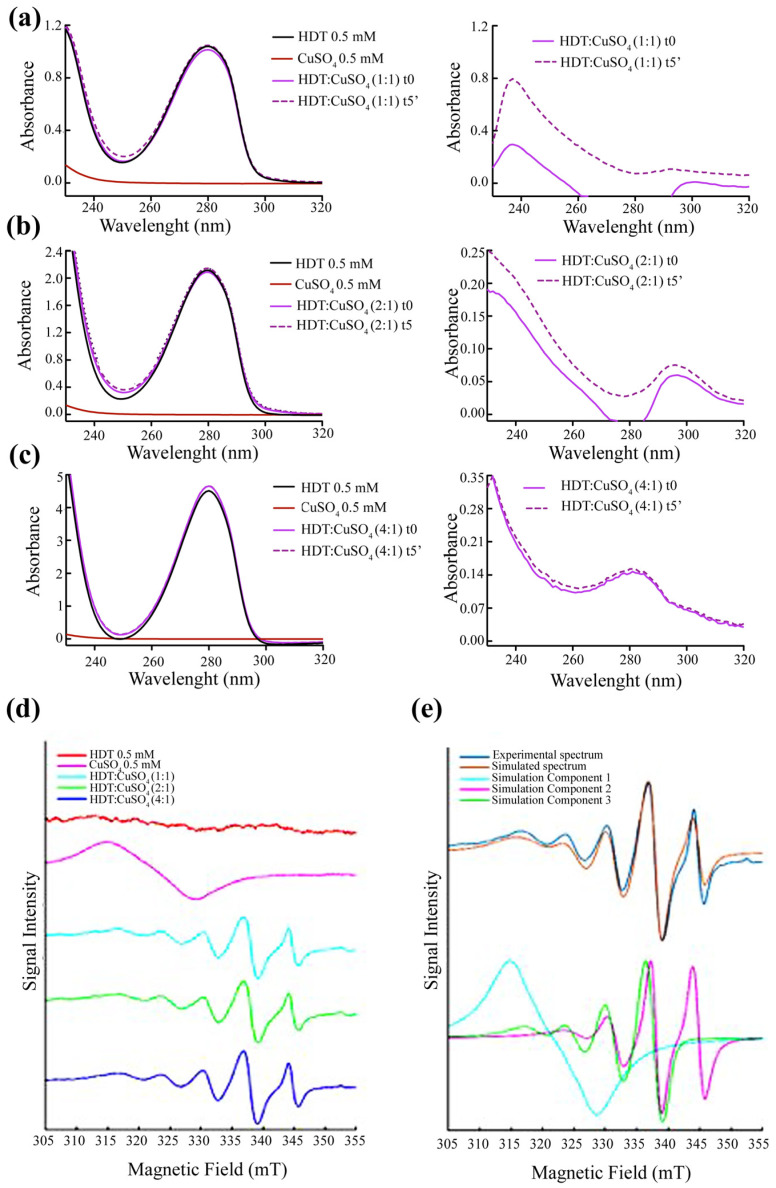
Changes in the UV and EPR spectra of CuSO_4_ upon addiction of HDT formation. Absorption spectra of HDT and CuSO_4_ at (**a**) 1:1 ratio, at time 0 and after 5 min of incubation at room temperature, one cm light path cuvette; (**b**) 2:1 ratio, at time 0 and after 5 min of incubation at room temperature, 0.5 cm light path cuvette, absorbance corrected; (**c**) 4:1 ratio, at time 0 and after 5 min of incubation at room temperature, 0.1 cm light path cuvette, absorbance corrected. In the right panels, the differential spectra are reported, obtained by subtracting the absorbance of HDT from that of the HDT:copper mixture. The stock solutions were prepared in ddH_2_O, as were the measurements in cuvettes. (**d**) Experimental EPR spectra (top to bottom): HDT in 1 mM aqueous solution (red); CuSO_4_ in 0.5 mM aqueous solution (magenta); HDT:copper at molar ratios of 1:1 mM (cyan), 2:1 mM (green), and 4:1 mM (blue). (**e**) EPR spectra of HDT:copper = 4:1 mM: the upper spectra show the experimental (blue) and the simulation (red) spectra; the lower spectra are the three simulation components described in the text.

The copper-chelating properties of HDT were preliminarily investigated through optical spectroscopy and EPR. We analyzed the variation of the optical spectrum of HDT upon the addition of copper sulfate (CuSO_4_). Specifically, we performed the optical spectra in the presence of a HDT:copper ratio of 1:1 (Figure 5a), 2:1 (Figure 5b), and 4:1 (Figure 5c). Although we did not observe any change in the visible region, indicative of a d-d transition, due to the low concentration of both ligands, we found an increase in the UV region of the absorbance spectrum, attributable to a ligand-to-metal charge transfer (LMCT) transition. In particular, we observe the appearance of a peak both in the 2:1 ratio (HDT:copper), between 280 and 320 nm, and in the 4:1 ratio (HDT:copper), between 260 and 300 nm. We repeated the spectra after 5 min of incubation of HDT and CuSO_4_, at room temperature (continuous line), obtaining no significant difference. The data obtained suggested the formation of a stable HDT-Cu with the possible stoichiometry of 2:1 and 4:1.

To deepen our investigation on the possible formation of the HDT–copper complex, we performed an EPR analysis. Figure 5d shows the experimental spectra of HDT and CuSO_4_ in aqueous solution and the three spectra of HDT:copper at 1:1, 2:1, and 4:1 molar ratios. Figure 5e shows the experimental spectrum of HDT:copper, at a 4:1 ratio, together with the simulated spectrum. The spectrum simulations identified three components shown in Figure 5e: two characterized by the typical multi-line signal of copper complexes, and one assigned to copper in aqueous solution. The simulation for the copper complexes was performed using the ‘chili’ function, while the ‘garlic’ function was used to simulate the spectrum of copper in aqueous solution. The parameters obtained from the fitting procedure are: g = 2.196, A = 103 MHz (simulation component 1); g = [2.09; 2.09; 2.27], A = [65; 65; 410] MHz (simulation component 2); and g = [2.09; 2.09; 2.146], A = [60; 60; 460] MHz (simulation component 3).

Component 1 described copper in aqueous solution, as described by other authors [52]. The assignment of components 2 and 3 can be done by analyzing the values of g_z_ and A_z_. A higher g_z_ value indicates a stronger axial bond. Based on the literature data, the g_z_ values found here are compatible with a square planar or tetrahedral geometry, but not with an octahedral configuration. In particular, component 3, with a lower g_z_ value, indicates a stronger ligand field in the equatorial plane, while component 2, with a higher g_z_, can be assigned to a complex with a tetrahedral geometry [51]. We, therefore, assumed that component 2 represents a square planar configuration, while component 3 represents a distorted square planar configuration, closer to a tetrahedral geometry. In both configurations, two HDTs should bind copper in the equatorial plane, whereas an octahedral configuration with hexa-coordination can be ruled out.

### 3.6. Description of the HDT–Copper Coordination Complexes by Molecular Modeling

**Figure 6:**
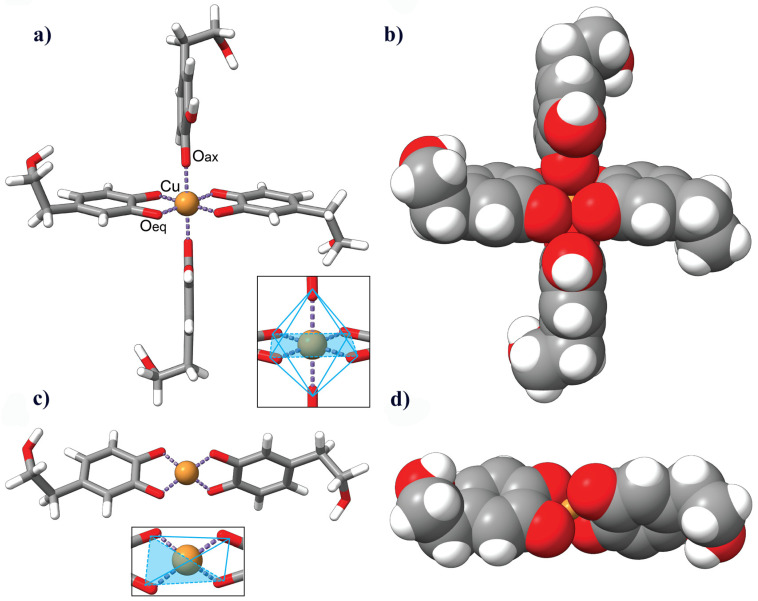
Molecular models illustrating the coordination of HDT molecules with a copper ion. (**a**,**c**): Stick models of four and two HDT molecules (standard heteroatom color coding) coordinating a copper ion (gold), respectively. The resulting chelation complexes display a hexa-coordinated structure in (**a**), with four equatorial Cu-O (Oeq) and two axial Cu-O (Oax) metal coordination bonds (depicted in purple), and a tetra-coordinate structure in (**c**) with four Cu-O bonds of similar distances. (**b**,**d**): Van der Waals representations of the corresponding coordination complexes, showcasing the three-dimensional arrangements of atoms. Insets in (**a**,**c**) depict the overall structures of the complexes, with (**a**) illustrating the octahedral Jahn–Teller distortion in the hexa-coordinated complex and (**c**) showcasing the slightly distorted square planar configuration in the tetra-coordinate complex.

Based on this evidence, we computationally modeled the coordination of copper with two or four molecules of HDT. The resulting chelation complexes exhibited distinct coordination environments. In the 2:1 complex, the central copper atom Cu (II) was found to be tetra-coordinated, while in the 4:1 complex, it was hexa-coordinated. The octahedral environment in the 4:1 complex displayed Jahn–Teller distortion, with the axial Cu-O bond being longer than the two remaining bonds in the equatorial plane (Figure 6a,b). On the other hand, the slightly distorted square planar configuration of the 2:1 complex featured uniform Cu-O bond distances of approximately 1.9 Å (Figure 6c,d).

To gain insights into the relative stability and energetics of the complexes, we determined the energy per mole (kJ/mol) using the Avogadro Auto-Optimize tool. The calculated potential energy values serve as indicators of the stability of the molecules. The 4:1 complex exhibited a potential energy of 572.856 kJ/mol, suggesting a higher energy state and potentially lower stability. In contrast, the 2:1 complex had a lower potential energy of 315.966 kJ/mol, indicating a comparatively more stable configuration.

## 4. Discussion

Copper is a transition metal acting as a cofactor as well as an allosteric regulator of enzymes involved in several essential cellular functions, including mitochondrial respiration, antioxidant defense, extracellular matrix remodeling, and cell migration. Nevertheless, the dysregulation of copper homeostasis is associated with the onset of various diseases, including cancer [53]. In recent years, a bulk of evidence has demonstrated that copper requirement is higher in cancer cells than in healthy cells, as also confirmed by the increased copper levels observed in serum and tumors of cancer patients [48]. Furthermore, copper is involved both in the process of angiogenesis and in the epithelial to mesenchymal transition (EMT), crucial steps for tumor growth and for the colonization of tissues distant from the primary tumor site [19,54]. This evidence has suggested the possible use of copper chelators, in association to classic chemotherapy agents, as a valid anti-tumor strategy [55]. However, the mechanisms through which cuproproteins/enzymes modulate tumor progression, and specifically the EMT, is still largely unknown.

In the field of chemotherapeutic agents, and, in particular, of copper chelators, there has been a growing interest in the anti-cancer properties of compounds of a natural origin. Previous studies conducted in our laboratory have shown that oleuropein, a polyphenolic compound belonging to the group of secoiridoids, contained in the leaves and fruits of the olive tree *Olea europaea*, is able to bind copper, thus suggesting its possible antitumor activity, and that it is more toxic towards cancer cells than towards their parental counterparts [25]. When consumed, oleuropein undergoes metabolism in the gastrointestinal tract and the liver: β-glucosidase enzymes catalyze the enzymatic hydrolysis of the glycosidic bond that connects glucose to the phenolic portion of the molecule, leading to the formation of oleuropein aglycon. Oleuropein aglycon can be further metabolized through various enzymatic reactions, such as hydroxylation, glucuronidation, and sulfation, resulting in the formation of hydroxytyrosol (HDT) and its conjugated forms. HDT is rapidly absorbed into the bloodstream and distributed throughout the body [56]. Indeed, it has been shown in Jurkat cells that HDT protects cells from the H_2_O_2_-induced impairment of intracellular iron homeostasis, subsequent DNA damage, and apoptosis by chelating the labile iron pool [57]. In addition, it has been demonstrated that HDT protects erythrocytes from mercury-induced oxidative stress [58]. Thus, based on this evidence and on a previous paper showing the copper-complexing properties of the HDT precursor oleuropein [25], we decided to investigate whether HDT could impair cancer cells by forming a complex with copper. To test our hypothesis, we chose three different triple-negative breast cancer (TNBC) cells (MDA-MB-231, MDA-MB468, and SUM159), due to their high aggressiveness and propensity to metastasize. Our first evidence showed that the HDT treatment of MDA-MB-231 and MDA-MB468 cells specifically induced the modulation of two well-known intracellular copper sensors: CCS [47], following 48 h treatment, and CcO [59], at 72 h HDT exposure, suggesting that HDT can perturb intracellular copper homeostasis. We also confirmed in these cell lines the modulation of the copper homeostasis prompt by HDT by an RT-qPCR analysis of CCS and of the copper-dependent protein required for extracellular matrix remodeling LOXL2. We found that 48 h of treatment with HDT induced, in both cells, an upregulation of the transcription levels of CCS, which corresponds to a reduced intracellular copper content. In contrast, LOXL2 mRNA was reduced in MDA-MB-231 and upregulated in MDA-MB-468. This different behavior in the two TNBC cell lines could be related to a differential response to the drug treatment that, in some cases, could initially push cells towards the activation of a resistance mechanism to the treatment itself. We did not observe any fluctuation in the levels of CCS and CcO in the SUM159. Of note, this cell line was also characterized by a reduced sensitivity to HDT, as demonstrated by its IC_50_ value for HDT, higher in comparison to that determined in the MDA-MB-231 and MDA-MB-468 cells. This lack of response to HDT could be related to a higher copper content of this cell line or to an intrinsic resistance to the dysregulation of copper homeostasis. Thus, the SUM159 cells have not been considered for long in our study.

Given the prominent role of copper in the modulation of kinases involved in the EMT [19], we found that the dysregulation of intracellular copper bioavailability, upon HDT treatment, was paralleled by the modulation of EMT hallmarks. In particular, through Western blot and immunofluorescence and RT-qPCR assays, we found the downregulation of the mesenchymal marker fibronectin and vimentin, and also of the epithelial markers E-cadherin and occludin, persisting, in some cases, for up to 72 h of HDT treatment. In MDA-MB-231 cells, after 48 and 72 h of treatment with HDT, these findings were further corroborated by the reduction of the transcript levels of most of the EMT transcription factors. Despite the different HDT-induced modulation of the TNBC cells’ epithelial and mesenchymal traits, in both cases, these converged into a significant decrease in their metastatic potential, as demonstrated through both the wound healing and Boyden chamber assays. The contemporary alteration of both the mesenchymal and epithelial tracts is not surprising. A growing body of data in the literature highlights the major role exerted by the hybrid mesenchymal/epithelial phenotype in driving cancer cell dissemination, as opposed to the fully mesenchymal phenotype [60,61,62]. This implies that the combination or transition between the two cell states may have more biological relevance than the complete transition to either state by itself.

To understand the mechanism by which copper chelation prompted by HDT could modulate the EMT and to specifically link the effects observed to the HDT’s copper-chelating features, we assessed the activation level of one of the copper-dependent kinases mainly involved in the EMT: AKT. In detail, we tested the levels of phosphorylated PDK1 and of phosphorylated AKT on the Ser473 residue. It is known that PDK1 is a copper-dependent kinase responsible for the phosphorylation of AKT at the Thr308 residue [63], positioned in the AKT activation loop. Thus, the Western blot analysis of the PDK1 phosphorylation levels allowed us to also evaluate indirectly the level of AKT phosphorylation on the Thr308 residue. However, to fulfill the maximum activation, AKT also required the phosphorylation of the Ser473 residue, located in its hydrophobic motif [64]. Of note, we have recently demonstrated that copper homeostasis is able to modulate the phosphorylation of AKT in Ser473 by a mechanism that has not been described yet [51]. In agreement with the data shown so far, we found a significant and persistent reduction over time of both PDK1 and AKT (Ser473) phosphorylation in MDA-MB-468. In MDA-MB-231, we found a significant downregulation of phosphorylated PDK1 only after 48 h exposure to HDT. However, this reduction of PDK1 phosphorylation observed in MDA-MB-231 was paralleled by a strong downregulation of all of the EMT-TFs mRNAs.

To finally establish whether the effects of HDT on the modulation of copper homeostasis, and then on the EMT signaling cascades, are related to the possible formation of an HDT–copper complex, we performed UV-spectrophotometric assays by incubating different HDT:copper molar ratios: 1:1, 2:1, and 4:1. The 1:1 molar ratio did not produce any change in the absorbance spectra of HDT, probably due to the low ligands concentration, whilst we observed a peak in the UV regions, suggesting a ligand-to-metal charge transfer (LMCT) transition, when HDT and copper were incubated in the molar ratio of 2:1 and 4:1, indicative of a possible change in the geometry of copper related to the interaction with HDT.

The EPR data confirmed this change in the copper geometry. In fact, from the spectra simulations, the EPR spectra described a superposition of two signals with different EPR parameters at all HDT:copper ratios. The signals can be reasonably associated with the formation of two copper complexes in different environments. In particular, the value of the parameter g_z_ is different in the two signals, which indicates that the two complexes have different bond strengths, one in the equatorial planes and the other in a tetrahedral geometry. The two complexes can, therefore, possibly be assigned to different tetra-coordinate complexes, as suggested in the literature for other copper complexes [65]. Clearly, the assignment of the EPR spectrum to defined geometries or coordination requires a more in-depth study of the system carried out under different conditions (e.g., pH, solvent, or temperature), which was outside the scope of this work. The assignment we have made here is based on the similarity of the EPR spectrum parameters with data from the literature [52,66,67,68,69]. Our hypothesis is supported by the notion that the catechol moiety of the HDT acts as a bidentate ligand capable of tetra-coordinating the CuII ion, as demonstrated in previous studies [67].

Of note, the ability of copper to form complexes with polyphenols, assuming different conformations, is well-known. For example, curcumin can form a complex with copper with a stoichiometry of 1:1 and 2:1, depending on the specific experimental conditions and the ratio of the reactants used [70]. Finally, our results are in line with the EPR spectra previously reported by Pirker and their colleagues concerning CuII-EGCG complexes [68]. Their observations included the contribution from two complexes which were clearly visible under physiological conditions, mirroring our experimental conditions and thereby reinforcing the consistency of our research with their work [68].

Spectroscopy data were corroborated by in silico prediction analysis. Indeed, HDT contains an ortho-hydroxy moiety, which exhibits a strong affinity for divalent metal ions [57]. Previous studies have demonstrated that CuII can be coordinated by two equatorial bidentate ligands, resulting in four equidistant bonds and adopting a distorted square planar configuration, as already found in other studies [25,66,71]. Moreover, a coordinatively saturated CuII center can adopt a six-coordinate configuration with two equatorial bidentates and two monodentate axial ligands. The coordination of six oxygen donors to the CuII ion forms an energetically favorable complex, known as a Jahn–Teller distorted octahedral model. This coordination pattern, characterized by 4 + 2 coordination bonds, features longer bonds on the axial donors. Conversely, when CuII forms a complex with four oxygen donors, it adopts a typical square planar model [72,73].

In the Jahn–Teller distorted octahedral model of the CuII ion, four strongly bound atoms occupy the equatorial positions at approximately 1.9 Å, while two atoms reside in the axial positions, with the longest bond distance measuring around 2.1 Å [42,74]. The most stable phenoxyl radicals of HDT are obtained by removing the hydrogen atom from the phenolic OH group in position 1 (HDT · rad), which retains a geometrical arrangement like the parent HDT molecule (Supplementary Figure S1a,b,e) [75]. Therefore, we assumed that the axial HDT molecules coordinate the CuII ion through their oxygen atoms in position 1. On the other hand, the equatorial HDT ligands coordinate the central CuII ion through their ortho-hydroxy moiety. The bond distances between CuII and the oxygen atoms in the tetra-coordinated configuration were expected to be relatively uniform [25,71]. Therefore, based on the results of molecular modeling and spectroscopic assays, we propose a model in which the HDT:copper molar ratio of 2:1 represents the tetra-coordinated complex which can exist in a planar or tetrahedral configuration, and, with less probability, a hexa-coordinated complex in which the HDT:copper molar ratio is 4:1. Moreover, the energy calculated suggested a higher stability of the 2:1 HDT:copper complex, in accordance with the experimental results, further supporting the validity of our findings [70,71]. The calculated energy values, besides representing relatively important information about the stability of the complexes, also serve as a foundation for the further analysis of the molecular properties, reactions, and stability within the context of this study. These insights contribute to a deeper understanding of the coordination behavior between HDT and copper, guiding future investigations and applications in various fields.

## 5. Conclusions

Overall, our data suggest that HDT could exert its effects on the EMT, resulting in the reduction of the metastatic behavior of TNBC cells, through its copper complexing ability. These findings further corroborate the EMT requirement of copper to accomplish its task in driving cancer cells towards metastasization. However, one of the main challenges and limitations in the use of polyphenols as chemotherapy adjuvants consists of their poor bioavailability due to their high metabolism rate and quick bodily removal [76]. Thus, the research is now focused on the synthesis of polyphenol-derived compounds to harness their beneficial effects on human health while overcoming this issue [77,78,79].

## Figures and Tables

**Table 1:** Primary antibodies used for Western blot analysis.

Primary Antibody	Origin	Company	Diluition
E-cadherin	Mouse	BD Transduction Laboratories(Milan, Italy) #610181	1:1000
Fibronectin	Rabbit	Merck Life Science S.r.l #F3648	1:3000
CCS	Rabbit	Santa Cruz Biotechnology (Dallas, TX, USA) #517412	1:1000
Subunit II complex IV	Mouse	Molecular Probes (Eugene, OR, USA) #A-6404	1:1000
ERK1/2	Rabbit	CellSignaling #4695	1:1000
Phospho ERK1/2 (Thr202/Tyr204)	Rabbit	CellSignaling #9101	1:1000
AKT	Rabbit	CellSignaling #4691	1:1000
Phospho-Akt (Ser473)	Rabbit	CellSignaling #4058	1:1000
GAPDH	Rabbit	Merck Life Science S.r.l #G9545	1:2000
Vinculin	Mouse	Santa Cruz Biotechnology # sc-5286	1:3000
Tubulin	Mouse	Santa Cruz Biotechnology #25336	1:1000

**Table 2:** Primers used for Real-Time PCR.

Gene	Primers
*SNAI1*	F: 5′-CCAGTGCCTCGACCACTATG-3′ R: 5-CTGCTGGAAGGTAAACTCTGG-3′
*SNAI2*	F: 5′-CCAAGCTTTCAGACCCCCAT-3′ R: 5′-GAAAAAGGCTTCTCCCCCGT-3′
*TWIST*	F: 5′-GCTTGAGGGTCTGAATCTTGCT-3′ R: 5′-GTCCGCAGTCTTACGAGGAG-3′
*ZEB1*	F: 5′-CAGCTTGATACCTGTGAATGGG-3′ R: 5′-TATCTGTGGTCGTGTGGGACT-3′
*MEMO1*	F: 5′-GCCGGAGTTTGTGGTGATTG-3′ R: 5′-CATTCAGCTGCGGTCCTGAG-3′
*LOXL2*	F: 5′-TACAAGCCAGAGCAACCCCT-3′ R: 5-CAGTGACTGCCTCTTTGGCA-3′
*ATOX1*	F: 5′-TGGTGGTATTGACGGTGTG-3′ R: 5′-CGTGATCAGAACCACGTCCA-3′

## Data Availability

The raw data obtained and analyzed during the current study are available from the corresponding authors upon reasonable request.

## Supplementary Materials

The following supporting information can be downloaded at: https://www.mdpi.com/article/10.3390/biology12111437/s1, Figure S1: Molecular geometry of (**a**) Hydroxytyrosol (HDT) and (**b**) its phenoxyl radical (HDT·rad) molecules optimized with B3LYP/6-31G (d, p) DFT method. The numbering corresponds to that reported by Davalos et al. (2018) [75].
