# Supplementary material for: Hydroxytyrosol Counteracts Triple Negative Breast Cancer Cell Dissemination via Its Copper Complexing Properties
Source: Biology (Basel). 2023 Nov 16;12(11):1437. doi: 10.3390/biology12111437 (PMC10669715; doi:10.3390/biology12111437)
Supplement: Supplementary file 1 [file biology-12-01437-s001.zip › biology-2657952-supplementary.pdf]

biology MDPI

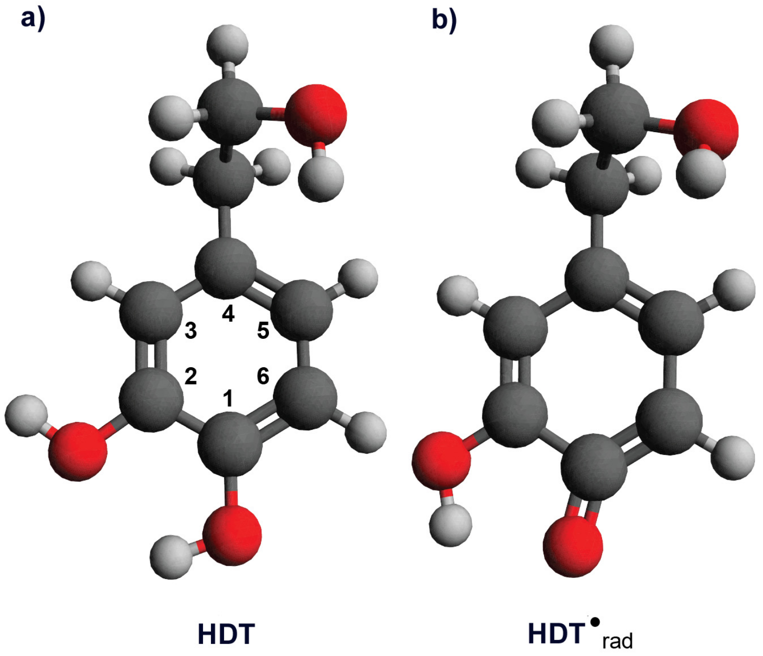

Supplementary Figure S1. Molecular geometry of (**a**) Hydroxytyrosol (HDT) and (**b**) its phenoxyl radical (HDT·rad) molecules optimized with B3LYP/6-31G (d, p) DFT method. The numbering corresponds to that reported by Davalos et al. (2018) [75].
